# Epidemiological, Clinical and Antiretroviral Susceptibility Characterization of Human Immunodeficiency Virus Subtypes B and Non-B in Pernambuco, Northeast Brazil

**DOI:** 10.1371/journal.pone.0155854

**Published:** 2016-05-24

**Authors:** Kledoaldo Lima, Élcio de Souza Leal, Ana Maria Salustiano Cavalcanti, Daniela Medeiros Salustiano, Luzidalva Barbosa de Medeiros, Sirleide Pereira da Silva, Heloísa Ramos Lacerda

**Affiliations:** 1 Postgraduate at Department of Tropical Medicine, Federal University of Pernambuco, Recife, Pernambuco, Brazil; 2 Institute of Biotechnology, Federal University of Pará, Belém, Pará, Brazil; 3 Sector of Virology, Central Public Health Laboratory of Pernambuco, Recife, Pernambuco, Brazil; 4 Department of Clinical Medicine, Federal University of Pernambuco, Recife, Pernambuco, Brazil; Fudan University, CHINA

## Abstract

**Background:**

HIV-1 diversity causes important differences in the virus’ biological properties and their interactions with hosts, such as cell tropism, responses to antiretroviral therapy, drug-resistance, and disease progression.

**Objectives:**

We evaluated the interrelationship of phylogenetic inference with epidemiological and laboratory data for HIV-1 isolates circulating in Pernambuco, Northeast Region—Brazil.

**Study design:**

A total of 168 HIV-1 *pol* sequences were analysed, 64 were obtained from 2002–2003, and 104, from 2007–2009. Socio-demographic, clinical, and behavioural data were obtained from medical records. Laboratory testing enabled the determination of recent HIV-1 infections and co-infections with HBV, HCV, HTLV, or syphilis. Surveillance drug-resistance mutation analysis and antiretroviral susceptibility profiling were performed using HIV Drug-Resistance Database.

**Results:**

HIV-1 non-B was associated with female, lower education, lower viral loads, and higher T cell counts mean. Frequencies of co-infection HIV-HBV, HIV-HCV, and HIV-syphilis were 27.8% (95% CI: 19.8–37.7), 1.04% (95% CI: 0.05–5.00) and 14.7% (95% CI: 8.6–23.0), respectively. Drug-resistant mutations rate was 2.98% (95% CI: 1.10–6.47). HIV-HBV subtype B co-infection was associated with men who have sex with men (MSM), higher education, higher viral loads and males. HIV-syphilis subtype non-B co-infection was associated with MSM status, lower T cell counts and males.

**Conclusions:**

Data showed the importance of molecular characterisations of the HIV-1 epidemic and its relation with epidemiological and clinical characteristics of the population, as well as its association with other infectious diseases, so they can effort to improve preventive measures for health services and more information about the progress and effects of the epidemic in Northeastern–Brazil.

## Introduction

HIV-1 is classified into 4 major groups: the major group (M), the non-major or non-outlier groups (N), the outlier group (O), and group P [1], which shows high genetic variability [2]. Group M is the predominant group throughout the world, it is responsible for the majority of global pandemic [1, 3].

The HIV-1 genetic variability is related to several factors: the lack of fidelity of the reverse transcriptase (RT) that causes a high mutation rate, a high replication rate *in vivo*, and the potential for genetic recombination in cells co-infected with different viral strains [4–6]. Another factor that influences genome variability is the process of diversity bottlenecking that occurs during transmission to a new host, in which the virus progeny produced early in infection are derived from the expansion of only a few viral particles that are successfully transmitted. Various factors interfere with viral transmission, such as selective pressure from the hosts’ innate immunity, the number of viral particles representing a particular genetic variant, the target cell density and the viral tropism for the CCR5 and CXCR4 co-receptors [7–8]. The increase in human population-migration rates also favours the continual expansion of viral diversity [9].

The high HIV-1 diversity causes important differences in the biological properties of the viral variants and their interactions with hosts [10]. For example, differences between variants occur in terms of evolutionary adaptation [11], mutation rates, cell tropism [10], responses to antiretroviral therapy [12], acquisition of drug-resistance [13–15], levels of baseline T cell counts before antiretroviral therapy [14], disease progression [16], response to vaccines [17], and transmission rates between individuals in different population-exposure categories [18–20].

A combination of phylogenetic analysis of HIV-1 with demographics, route of infection, clinical and laboratory data provides a better understanding of a local epidemic and measures for developing control and prevention strategies, because epidemiological trends can be observed through molecular analysis, justifying sustained focus in prevention on higher risk groups, for example, Chalmet et al. (2010) observed that subtype B was associated with MSM and Caucasians [21]. Furthermore, recombination events have a tendency to be more frequent in the *gag/pol* regions [22].

Most studies linking phylogenetic and epidemiological data contain little information related to HIV-1 subtype F, whose prevalence is considered high (~22–37%) in Pernambuco, Northeast Brazil [23–25]. Thus, we evaluated the interrelationship of phylogenetic data with epidemiological and laboratory data pertaining to HIV-1 subtypes that are currently circulating in Pernambuco, Northeast—Brazil.

## Materials and Methods

### Study population

We examined 168 HIV-1 *pol* sequences obtained from 2 previous studies [24–25]. Sixty-four sequences were obtained from samples collected from patients followed at the Hospital of the Federal University of Pernambuco (Pernambuco, Northeast—Brazil) in 2002–2003 [24], and 104 sequences were obtained from individuals who seeking to any of the 5 largest voluntary counselling and testing centres (VCTs) of Pernambuco, during 2007–2009 [25]. The individuals enrolled in those studies were diagnosed with HIV-1 infection according to the recommendations of Brazil’s Ministry of Health, and were antiretroviral-drug naive. Sociodemographic and laboratory information was obtained from medical records. In addition, serological assays were performed to detect co-infection with hepatitis B virus (HBV), hepatitis C virus (HCV), human T lymphotropic virus (HTLV), and syphilis in the samples collected during 2007–2009. Data from the BED-CEIA assay were available only for samples obtained from 2007–2009. All participants provided written consent to collect their blood and sequencing the viral genome. All items of this study was approved by the Ethics Committee on Health Sciences Centre of the Federal University of Pernambuco (CCS-UFPE) under number 114,722.

### Laboratory diagnosis of co-infections

The laboratory diagnosis of HBV, HCV, HTLV, and syphilis was performed with 97/104 (93.3%) of the samples from 2007–2009. Blood samples were collected in EDTA tubes and after centrifugation, plasma aliquots were separated and stored at −70°C. Diagnoses of present and/or past HBV infection was performed by serological assays detecting HBsAg and total anti-HBc (Architect System, Abbott Diagnostic Division, Ireland). Serological screening for anti-HCV was performed to detect HCV infection (Architect System, Abbott Diagnostic Division, Ireland), and HCV-RNA quantification was performed on antibody-positive samples using the Cobas AmpliPrep and the Cobas TaqMan HCV assay (Roche Diagnostic, Germany). To determine the presence of syphilis, serological diagnosis was performed with a treponemal assay on the Architect System. Posteriorly, positive samples were retested with a non-treponemal assay (Venereal Disease Research Laboratory, Wiener Lab, Argentina). In cases where conflicting results were observed, the samples were reanalysed with an independent treponemal assay (TPHA Syphilis Assay, HUMAN Diagnostic, Germany), as guideline by Brazil’s Ministry of Health (http://www.aids.gov.br/pagina/regulamentacao-de-tests; http://www.cdc.gov/Mmwr/preview/mmwrhtml/mm5732a2.htm). The HTLV assays was performed using the Architect System.

### Sequencing of the HIV-1 polymerase gene (*pol*)

All technical procedures for sequencing HIV-1 polymerase gene have been described in previous papers whose samples were sequenced [24–25]. A 918-base pair fragment was obtained comprising the entire *PR* and *RT* sequences, corresponding to positions 2262–2549 from *PR* and 2661–3290 from *RT* of the reference strain HXB2 (GenBank Accession Number K03455).

Alignments of sequences were performed using Clustal X software [26], followed by manual editing with BioEdit software [27]. Viral subtyping was determined using the REGA Automated Tool for HIV-1 & 2 subtyping (Version 2.0) (http://www.bioafrica.net/rega-genotype/html/subtypinghiv.html) and phylogenetic inference with Neighbor-Joining and Maximum Likelihood methods were performed using MEGA5 software.

### Antiretroviral resistance analysis

Surveillance Drug-Resistance Mutations (SDRMs) were identified using the HIV Drug-Resistance Database of Stanford University (http://cpr.stanford.edu/cpr.cgi) and the standard SDRMs list established by the World Health Organization (WHO) [28]. Susceptibility to antiretrovirals were analysed using the HIVdb Program of Stanford University (http://sierra2.stanford.edu/sierra/servlet/JSierra).

### Statistical analysis

The Pearson chi-squared and Fisher's exact test were used for the analysis of categorical variables. The Kruskal–Wallis test was applied for the comparison of medians with continuous variables. Stepwise logistic regression was performed to select the most significant variables. Variables with a moderate association (p ≤ 0.2), whereas a threshold of p ≤ 0.05 was applied for the stepwise elimination of variables considered as risk factors. Stratified analyzes by calculating the odds Mantel-Haenzel were performed to evaluate the interaction between the viral subtype and status for HBV and syphilis infections with other variables. Data were analysed using STATA V.13 software (Stata Corp., USA).

## Results

### Epidemiological and laboratory differences between HIV-1 subtypes B and non-B

We evaluated 168 viral sequences, of which 103 query sequences (61.3%) were subtype B, 53 subtype F (31.5%) and two HIV-1 C (1.2%). Ten query sequences (6%) were unique recombinant forms (8 BF, 1 AG, and 1 BC).

We observed the occurrence of a higher frequency of subtype B in males and individuals with higher education (Table 1). Statistically significant associations were also observed between the subtype B and higher viral loads and lower T cell counts. In a multivariate model, males (p = 0.054) and viral load above 100,000 copies/ml (p = 0.065) were more common in patients infected with subtype B, however with statistical significance borderline.

**Table 1:** Demographic and laboratory characteristic among HIV-1 subtypes B and non-B.

Variables	Subtipe B (n = 103)	Subtipe Non-B^¶^ (n = 65)	Univariate analysis	
			OR (CI—95%)	p-value
**Gender (n = 167)**				
Male	66 (64.7%)	31 (47.7%)	1.0	-
Female	36 (35.3%)	34 (52.3%)	2.01 (1.02–3.99)	0.029^†^
**Age (years) (n = 167)**	34.0 ± 10.6	34.6 ± 10.4	0.995 (0.966–1.025)	0.762
**Education (n = 143)**				
< 8 years	50 (56.2%)	40 (74.1%)	1.0	
≥ 8 years	39 (43.8%)	14 (25.9%)	0.45 (0.21–0.93)	0.033^†^
**Marital status (n = 67)**				
Single	24 (47.1%)	9 (56.3%)	1.0	-
Married	19 (37.3%)	6 (37.5%)	0.85 (0.28–2.60)	0.773
Divorced	3 (5.9%)	0 (-)	Not calculed	-
Windowed	5 (9.8%)	1 (6.2%)	Not calculed	-
**Pregnancy (n = 71)**				
Yes	4 (10.8%)	5 (14.7%)	1.0	-
No	33 (89.2%)	29 (85.3%)	1.42 (0.35–5.80)	0.623*
**Exposure category (n = 167)**				
HTS	68 (66.7%)	52 (80%)	1.0	-
MSM	34 (33.3%)	13 (20%)	0.5 (0.22–1.10)	0.062
**Infections status (n = 101)**				
Recent	12 (22.2%)	9 (19.1%)	1.0	-
Long-term	42 (77.8%)	38 (80.9%)	0.83 (0.31–2.18)	0.704
**CD4 count (median)**◊	226.5 (78; 414)	341 (159; 488)	-	0.036^†^
**CD4 levels (n = 131)**				
< 200 cells/mm^3^	32 (39.0%)	13 (26.5%)	1.0	-
≥ 200 cells/mm^3^	50 (61.0%)	36 (73.5%)	1.77 (0.77–4.14)	0.145
**Viral load (median)**◊	65.000 (7.618; 194.000)	18.185 (1.470; 53.973)	-	0.023^†^
**Viral load levels (n = 138)**				
< 100.000 copies/ml	52 (60.5%)	40 (76.9%)	1.0	-
≥ 100.000 copies/ml	34 (39.5%)	12 (23.1%)	0.46 (0.20–1.06)	0.047^†^
**Co-infections**				
**HIV-HBV (n = 97)**				
Negative	35 (67.3%)	34 (77.3%)	1.0	-
Positive	17 (32.7%)	10 (22.7%)	1.65 (0.61–4.55)	0.279
**HIV-HCV (n = 96)**				
Negative	52 (100%)	43 (97.7%)	1.0	-
Positive	(-)	1 (2.3%)	Not calculated	-
**HIV-HTLV (n = 91)**				
Negative	47 (100%)	44 (100%)	1.0	-
Positive	(-)	(-)	Not calculated	-
**HIV-Syphilis (n = 95)**				
Negative	44 (86.3%)	37 (84.1%)	1.0	-
Positive	7 (13.7)	7 (15.9)	0.84 (0.27–2.62)	0.765

HTS, Heterosexuais; MSM, Mens who have sex with men.

^**¶**^ Among the 65 HIV-1 subtypes non-B, 53 were subtipe F;

^†^Statistically significant association;

* Fischer’s exact test;

^**◊**^ Median (P_25_; P_75_)–Kruskal-Wallis test;

^(-)^ Not detected.

With respect to the exposure category, the subtype B was more common in men who have sex with men (MSM), however no statistic significant. When the variable “heterosexual” was partitioned between male and female heterosexuals, it was observed that approximately 50% of the women harboured a subtype non-B, compared to 29% of heterosexual males and 20% of MSM. However, in both cases, no statistical significance was observed.

For HIV-1 subtype F, which represented approximately 80% of non-B subtypes (53/65), there was a statistically significant association with a lower viral load (p = 0.0049), but no association with the median T cell count (p = 0.067), when they were analysed separately from subtypes non-B.

Samples tested for serological assays refer with 94/104 (93.3%) for the time of 2007–2009 and 94/168 (56%) of the all samples. The prevalence of present or past HIV-HBV co-infection was 27.8% (27/97) (IC95%: 19.8–37.7), with 4 samples showing positive results for HBsAg, 19 samples showing serological reactivity for anti-HBc alone, and 4 samples having reactivity against both HBsAg and anti-HBc. The 4 samples with isolated reactivity for HBsAg were classified as representing long-term infection by BED-CEIA testing. The prevalence of HIV-syphilis and HIV-HCV co-infections were, respectively, 14.7% (14/95) (IC95%: 8.6–23.0) and 1.04% (01/96) (IC95%: 0.05–5.00). No sample was positive for HTLV infection. The unique case of co-infection by HIV-HCV belonged to subtype F, which showed a high HCV viral load. Among the 101 samples collected in 2007–2009 that were evaluated for infection statuses by BED-CEIA assay, 21 (20.8%) were determined as recent infections.

**Fig 1:**
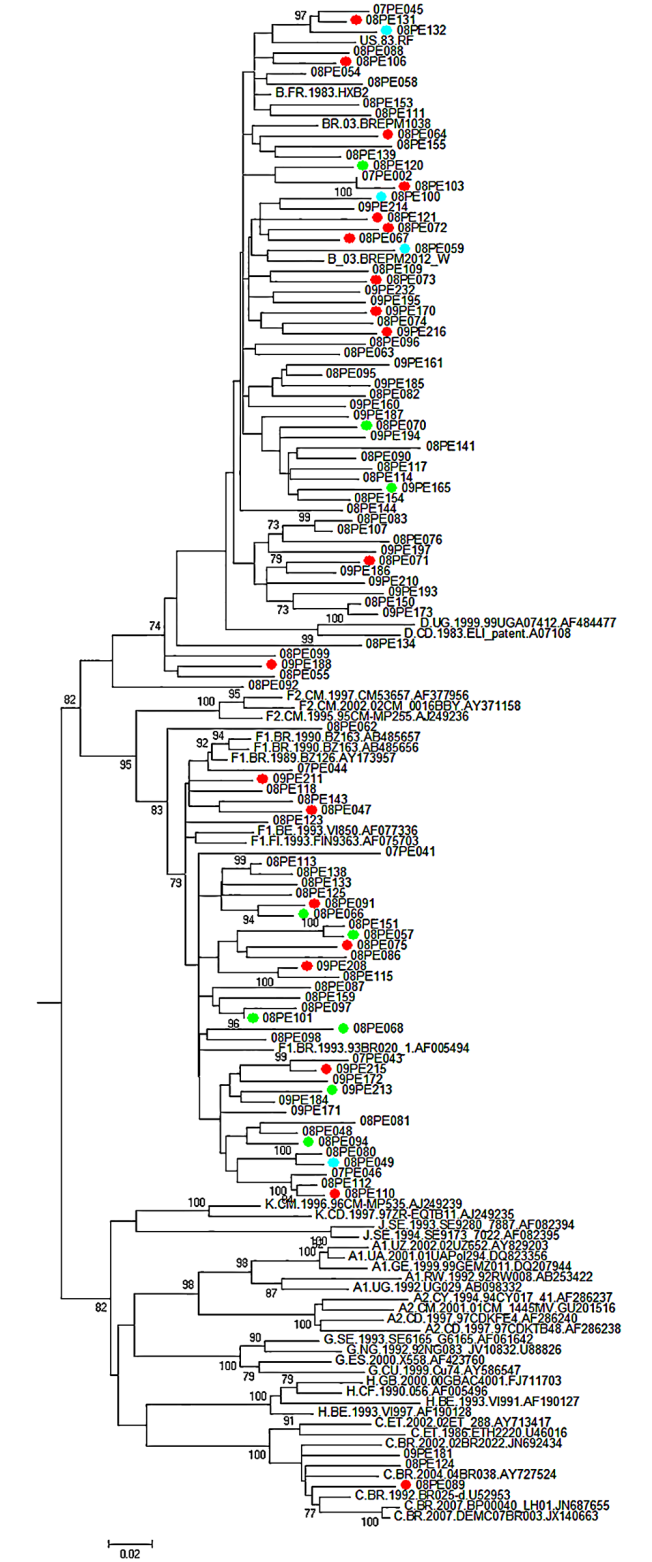
Phylogenetic analysis of sequences from 2007–2009. Bootstrap support values of ≥ 70 were considered significant and only bootstrap of 70 or higher are shown. red circles: co-infections HIV/HBV; Green circles = co-infections HIV/Syphilis; blue circles = co-infections HIV/HBV/Syphilis.

Phylogenetic analysis showed that a minority of sequences with co-infections grouped in clusters with good bootstrap (≥90), problably, there was no pattern of transmission for co-infected individuals (Fig 1).

We observed that the viral subtype presents interaction with HBV or syphilis infections and other clinical and epidemiological variables. However, it did not constitute confounding factor (Tables 2 and 3). HBV co-infection was associated with MSM, male gender, higher education, and higher HIV-1 viral loads. However, for males, the relationship between odds ratio crude and Mantel-Haenszel shows the observation of adjacent confounders that we would need more samples to determine this data. Co-infections with HIV-syphilis were associated with MSM, T cell counts less than 200 and males interacting with the non-B subtype.

**Table 2:** Stratified analysis of individuals with co-infecttion HBV/HIV-1 subtypes B and non-B.

	HIV-1 subtipe B			HIV-1 subtipe non-B			Stratified Statistical Analysis	
Variables	HBV+ n (%)	HBV- n (%)	p-value	HBV+ n (%)	HBV- n (%)	p-value	ORcrude	OR_MH_^ω^
**Exposure category (n = 95)**^§^	(n = 27)^¶^	(n = 70)^¶^		(n = 27)^¶^	(n = 70)^¶^			
MSM	10 (58.9)	08 (23.5)	0.014	01 (10)	06 (17.6)	0.56 *	2.65(1.00–7.01)	2.46(0.91–6.61)
HTS	07 (41.1)	26 (76.5)		09 (90)	28 (82.4)			
**T cells count (n = 96)**								
CD4 < 200	02 (20)	06 (22.2)	0.88 *	01 (14.3)	12 (23.1)	0.60 *	0.72(0.18–2.81)	0.72(0.18–2.87)
CD4 ≥ 200	08 (80)	21 (77.8)		06 (85.7)	40 (76.9)			
**Viral load**^μ^ **(n = 73)**								
< 100.000	06 (50)	24 (85.7)	0.018 *	06 (85.7)	22 (84.6)	0.94 *	0.29(0.09–0.98)	0.31(0.09–1.03)
≥ 100.000	06 (50)	04 14.3)		01 (14.3)	04 (15.4)			
**Gender (n = 96)**								
Male	15 (88.2)	20 (57.2)	0.026 *	03 (30)	17 (50)	0.27 *	1.73(0.68–4.08)	1.58(0.61–4.07)
Female	02 (11.8)	15 (42.8)		07 (70)	17 (50)			
**Education (n = 76)**								
< 8 years	04 (33.3)	20 (69)	0.037 *	04 (57.2)	19 (67.8)	0.59 *	0.34(0.12–0.98)	0.34(0.12–1.00)
≥ 8 years	08 (66.4)	09 (31)		03 (42.8)	09 (32.2)			

MSM, Men who have sex with men; HTS, Heterosexuais.

^¶^ Total of samples tested serologically for co-infection HBV-HIV;

^§^ Available data for each variable in the analysis;

* Fisher's exact test;

^χ^ MSM: Men who have sex with men;

^¥^ HTS: Heterosexuais;

^μ^ Viral load (copies/ml);

^ω^Odds ratio Mantel-Haenszel;

**Table 3:** Stratified analysis of individuals with co-infecttion Syphilis/HIV-1 subtypes B and non-B.

	HIV-1 subtipe B			HIV-1 subtipe non-B			Stratified Statistical Analysis	
Variables	Syphilis+ n (%)	Syphilis- n (%)	p-value	Syphilis+ n (%)	Syphilis- n (%)	p-value	ORcrude	OR_MH_^ω^
**Exposure category (n = 91)**^§^	(n = 14)^¶^	(n = 81) ^¶^		(n = 14)^¶^	(n = 81)^¶^			
MSM	02 (28.6)	15 (34.9)	0.77*	04 (57.1)	03 (8.8)	0.002 *	2.46(0.75–8.02)	2.41(0.77–9.90)
HTS	05 (71.4)	28 (65.1)		03 (42.9)	31 (91.2)			
**T cells count (n = 67)**								
CD4 < 200	-	07 (23.3)	0.23 *	03 (75)	04 (14.3)	0.007 *	2.13(0.46–9.90)	2.00(0.47–8.56)
CD4 ≥ 200	05 (100)	23 (76.7)		01 (25)	24 (85.7)			
**Viral load**^μ^ **(n = 72)**								
< 100.000	06 (100)	24 (75)	0.17 *	04 (100)	25 (83.3)	0.38	6.53(0.80–53.51)	6.57(0.72–59.67)
≥ 100.000	-	08 (25)		-	05 (16.7)			
**Gender (n = 94)**								
Male	03 (42.9)	30 (69.8)	0.17 *	06 (85.7)	14 (37.8)	<0.001	2.09(0.63–6.90)	2.15(0.75–6.09)
Female	04 (57.1)	13 (30.2)		01 (14.3)	23 (62.2)			
**Education (n = 74)**								
< 8 years	02 (33.3)	22 (66.7)	0.14 *	03 (75)	20 (64.5)	0.68 *	0.52(0.14–2.00)	0.54(0.14–2.03)
≥ 8 years	04 (66.7)	11 (33.3)		01 (25)	11 (35.5)			

MSM, Men who have sex with men; HTS, Heterosexuais.

^¶^ Total of samples tested serologically for co-infection HBV-HIV;

^§^ Available data for each variable in the analysis;

* Fisher's exact test;

^χ^ MSM: Men who have sex with men;

^¥^ HTS: Heterosexuais;

^μ^ Viral load (copies/ml);

^ω^Odds ratio Mantel-Haenszel;

### Profiles of antiretroviral resistance

Surveillance Drug Resistance Mutations (SDRMs) has been a prevalence of 2.98% (95%CI: 1.10–6.47), in 5/168 sequences analysed (Table 4). Only 1 patient (08PE082) showed resistance to 2 classes of antiretroviral drugs (NRTI + NNRTI) and one showed a high resistance limited to PI’s, with mutations L82A and L90M. The patient who had major mutations in the protease was co-infected with HBV. All patients with SDRMs were classified as having evidence of long-term infection by BED-CEIA assay.

**Table 4:** Drug resistance mutations and antiretroviral resistance profile at patients from Pernambuco, Northeast–Brazil.

Sequence/ Subtype	Gender/Exposure category/	PI major mutations	PI minor mutations	ITRN mutations	ITRNN mutations	Drug-resistance profile		
						Low	Intermediate	High
08PE165 B	F/HTS/PHIV+/ Syphilis	-	L10V^¶^ A71T^¶^	-	P225H	NFV NVP	EFV	-
08PE121 B	M/HTS/ HBsAg+	L82A L90M	L10F^¶^ E33F^¶^ K43T^¶^ A71V^¶^G73S	-	-	TPV/r	FVP/r LPV/r	ATV/r IDV/r NFV SQV/r
08PE082 B	M/HTS	-	-	V75L F77L M184V	K101E G190S	ABC DDI ETR	RPV	3TC FTC EFV NVP
09PE172 F	M/MSM	-	L10V^¶^	-	K103N	-	-	EFV. NVP
08PE055 BF	M/HTS	-	-	-	G190A	ETR RPV	EFV	NVP

PI, Protease inhibitors; NRTIs, Nucleoside reverse transcriptase inhibitors; NNRTI, non-nucleoside reverse transcriptase inhibitors; ABC, abacavir; ATV, atazanavir; DDI, didanosine; EFV, efavirenz; ETR, etravirine; FVP, fosamprenavir; FTC, emtricitabine; IDV, indinavir; PVL, lopinavir; NFV, nelfinavir; NVP, nevirapine; RPV, rilpivirine; SQV, saquinavir; 3TC, lamivudine; HTS, heterosexual; MSM, men who have sex with men; PHIV +, HIV + partner.

^¶^ Mutations not contained in the SDRM list established by the World Health Organization (WHO) (BENETT et al. 2009). However, according to the HIVdb Program—Stanford University for the evaluation of antiretroviral susceptibility, they may be associated with decreased susceptibility that when combined with other mutations or due to other intrinsic characteristics to each corresponding codon.

## Discussion

Data from 168 HIV-1 *pol* sequences were evaluated with respect to epidemiology, laboratory data, co-infections, and antiretroviral susceptibility. The local epidemic in Pernambuco is characterised by heterogeneity of HIV-1 subtypes, but with a predominating subtype B (60.9%). Subtypes non-B (n = 65) consisted mostly of HIV-1 F (n = 53, 81.5%).

Subtype B was associated with the following risk factors: males, higher education level, higher viral loads, and lower median T cell counts, compared with subtypes non-B. Unfortunately, due to the disadvantage of the cross-sectional design of the study, we could not confirm the association of HIV-1 subtypes non-B and the possible protection for disease progression. However, some authors suggest an association of the viral subtype with clinical progression [29–30], none of these studies included HIV-1 F, the major HIV-1 subtype non-B detected in our study. Although the individuals were from two different services health (hospital and VCTs), its epidemiological characteristics were evaluated together, and to avoid possibles biases we observed that all patients were treated in public health services (hospital or VCTs), which suggests that they were of the same socio-economic level; users were residents of the same region (very nearby cities) anddespite years of 2002–2003 have a larger number of MSM, after analysis of OR_MH_, sexual exposure category did not constitute confusion factor (data not shown). HIV-1 B was more frequent in MSM, without statistical significance, and considering that MSM had higher education than heterosexuals (p <0.0001), this may explain the association of higher education level with this subtype,. The heterosexual women showed the highest rate of HIV-1 non-B infection (48.6%), followed by heterosexual men (37.2%) and MSM (27.6%), confirming the findings of Geretti et al. [12] and Hawke et al. [31], which showed a high prevalence of subtypes non-B in heterosexuals, especially in women. Besides, Dias et al. [32] suggested a link between sexual transmission intercourse (vaginal or anal) with HIV-1 subtype.Wefound that occurred the expansion of subtypes non-B into the MSM, between 2002–2003 and 2007–2009 (p = 0.0019, data not shown). Almeida et al. [20] also showed an increase in subtypes non-B among MSM (particularly C and BC recombinants) in the southern region of Brazil, which reinforces the proliferation of non-B subtypes in the country, especially subtype C and BF recombinants [20, 33–34].

We detected a rate of 27.8% (95% CI: 19.8–37.7) for past or present co-infection with HBV (27/97), and 8 cases (8.2%) showed the presence of HBsAg. The percentages of co-infection with syphilis or HCV were respectively 14.7% (95% CI: 8.6–23.0) and 1.04% (95% CI: 0.05–5.00). In other studies with HIV-positive individuals in Pernambuco (Northeast–Brazil), a high prevalence also was found for co-infections with HIV-HBV, with frequencies of 10.3% and 38.7% for HBsAg and anti-HBc positivity, respectively [35]. For HIV-HCV co-infection, these frequencies have been reported as 10.7% [35] and 3.2% [36]. Salvador, another large city in Northeast—Brazil, has also been reported to have a large proportion of HIV-HBV co-infections (22%) and HIV-HCV co-infections (13%) [37]. Generally, lower frequencies for coinfections in our study may be explained by the fact that the above studies were performed with patients followed up in infectious disease-service centres at hospitals, whereas our study samples came from outpatients of VCTs who were newly diagnosed with HIV-1. Thus, a faster diagnosis of HIV infection could have served to promote increased awareness of prevention and control measures for new STIs. Our study showed a small identifying of clusters of possible transmission of co-infections. The establishment of the dynamics of transmission may be affected by various factors, as a low number of samples compared to the population or difficulties of phylogenetics analysis to identify clusters of transmission.

The results show a statistical interaction between subtype B and coinfection with HBV and other epidemiological and clinics variables, as higher education, lower T cells count, higher viral loads and MSM. Other studies conducted in Brazil also showed an association among co-infections HIV-HBV with males [38–39], MSM [38–40] and higher education level [38], although the analysis with HIV-1 subtyping were not performed and it is a differential of this study. It is interesting the association between individuals with higher education and co-infection HIV-1 B / HBV, because we expected the opposite due to greater awareness of preventive measures. However, MSM had higher educational level than heterosexuals (p <0.0001, data not shown), and those were associated with subtype B infection, therefore we can assume that MSM influenced the association between education and co-infection.

Syphilis coinfection was associated with MSM and lower median T cell counts presenting interaction with non-B subtypes. Although coinfection HIV-syphilis is a major public health problem in many countries, especially among MSM [41–42], we found a shortage of data about HIV-1 subtypes and its association with syphilis and HBV infection. Limiting factor for these analyses are that the type of co-infection was studied using a low number of available samples, which could have hidden a better understanding of these data. Another limitation of this study is the fact that, unfortunately, we did not get the results of serological testing for HIV-negative individuals and thus we can not determine whether the statistical associations also occur in HIV-negative individuals independent of co-infection.

The prevalence of SDRMs was 2.98% (95% CI: 1.10–6.47). Primary resistance to antiretrovirals was lower than that seen in another large city in Northeast—Brazil (Fortaleza) (9.5%) [43], as well as in Rio de Janeiro, where a high (~15%) drug-resistance rate was detected in newly diagnosed individuals at VCTs [44]. However, in both cities, subtype B represented 85 and 78% of the isolates, respectively. In contrast, our samples showed a significant percentage (38.7%) of subtypes non-B. Some studies have reported the greatest accumulation of antiretroviral resistance in HIV-1 B isolates [14–15] and an increased susceptibility of HIV-1 F to protease inhibitors [5]. Reinforcing these findings, a study by the National Network for the Surveillance of Drug Resistance in Brazil (HIV-BResNet) reported a lower level of resistance in Brazilian cities with the highest percentages of subtypes non-B, such as Porto Alegre and Salvador [45], which may suggest the existence of any relationship between the viral subtype and the acquisition of SDRMs.

we have identified epidemiological and laboratory peculiarities relating to circulating HIV-1 subtypes in the Northeast—Brazil. Additional controlled studies are necessary for a deeper understanding of the main epidemiological, laboratory, and viral characteristics that differentiate major subtype B viruses from the increasingly prevalent HIV-1 subtype F. The proportion of HIV-1 subtype F viruses is high in the Pernambuco, and our data therefore may provide important clues for improving infection prevention strategies.
