# Correction: Epidemiological, Clinical and Antiretroviral Susceptibility Characterization of Human Immunodeficiency Virus Subtypes B and Non-B in Pernambuco, Northeast Brazil

**DOI:** 10.1371/journal.pone.0158192

**Published:** 2016-06-27

**Authors:** Kledoaldo Lima, Élcio de Souza Leal, Ana Maria Salustiano Cavalcanti, Daniela Medeiros Salustiano, Luzidalva Barbosa de Medeiros, Sirleide Pereira da Silva, Heloísa Ramos Lacerda

The HIV-1 sequence data are missing from the published article. Please view these data as file S1 Data of this correction.

## Supporting Information

S1 Data HIV-1 sequence data. (DOCX)
