# Supplementary material for: Correction: Epidemiological, Clinical and Antiretroviral Susceptibility Characterization of Human Immunodeficiency Virus Subtypes B and Non-B in Pernambuco, Northeast Brazil
Source: PLoS One. 2016 Jun 27;11(6):e0158192. doi: 10.1371/journal.pone.0158192 (PMC4922594; doi:10.1371/journal.pone.0158192)
Supplement: S1 Data — (DOCX) [file pone.0158192.s001.docx]

BankIt1904654 08PE056 KX020958

BankIt1904654 08PE123 KX020959

BankIt1904654 09PE211 KX020960

BankIt1904654 02PE047 KX020961

BankIt1904654 08PE048 KX020962

BankIt1904654 08PE110 KX020963

BankIt1904654 09PE215 KX020964

BankIt1904654 02PE036 KX020965

BankIt1904654 02PE041 KX020966

BankIt1904654 07PE044 KX020967

BankIt1904654 07PE041 KX020968

BankIt1904654 09PE171 KX020969

BankIt1904654 02PE014 KX020970

BankIt1904654 03PE082 KX020971

BankIt1904654 02PE023 KX020972

BankIt1904654 08PE081 KX020973

BankIt1904654 08PE091 KX020974

BankIt1904654 09PE213 KX020975

BankIt1904654 08PE097 KX020976

BankIt1904654 03PE103 KX020977

BankIt1904654 08PE118 KX020978

BankIt1904654 08PE049 KX020979

BankIt1904654 07PE046 KX020980

BankIt1904654 08PE125 KX020981

BankIt1904654 08PE159 KX020982

BankIt1904654 08PE066 KX020983

BankIt1904654 08PE047 KX020984

BankIt1904654 08PE080 KX020985

BankIt1904654 02PE016 KX020986

BankIt1904654 08PE112 KX020987

BankIt1904654 09PE172 KX020988

BankIt1904654 08PE133 KX020989

BankIt1904654 02PE029 KX020990

BankIt1904654 09PE184 KX020991

BankIt1904654 08PE098 KX020992

BankIt1904654 08PE068 KX020993

BankIt1904654 08PE094 KX020994

BankIt1904654 02PE065 KX020995

BankIt1904654 02PE025 KX020996

BankIt1904654 07PE043 KX020997

BankIt1904654 08PE101 KX020998

BankIt1904654 03PE081 KX020999

BankIt1904654 08PE113 KX021000

BankIt1904654 08PE138 KX021001

BankIt1904654 08PE143 KX021002

BankIt1904654 08PE087 KX021003

BankIt1904654 02PE040 KX021004

BankIt1904654 02PE039 KX021005

BankIt1904654 08PE151 KX021006

BankIt1904654 09PE208 KX021007

BankIt1904654 08PE115 KX021008

BankIt1904654 08PE057 KX021009

BankIt1904654 08PE075 KX021010

BankIt1904654 08PE086 KX021011

BankIt1904654 08PE092 KX021012

BankIt1904654 08PE055 KX021013

BankIt1904654 08PE134 KX021014

BankIt1904654 09PE188 KX021015

BankIt1904654 08PE099 KX021016

BankIt1904654 03PE0104 KX021017

BankIt1904654 08PE062 KX021018

BankIt1904654 08PE124 KX021019

BankIt1904654 08PE089 KX021020

BankIt1904654 09PE181 KX021021

BankIt1904654 08PE111 KX021022

BankIt1904654 08PE058 KX021023

BankIt1904654 09PE194 KX021024

BankIt1904654 03PE083 KX021025

BankIt1904654 08PE095 KX021026

BankIt1904654 09PE216 KX021027

BankIt1904654 02PE008 KX021028

BankIt1904654 08PE063 KX021029

BankIt1904654 08PE083 KX021030

BankIt1904654 07PE045 KX021031

BankIt1904654 08PE131 KX021032

BankIt1904654 02PE044 KX021033

BankIt1904654 09PE185 KX021034

BankIt1904654 02PE051 KX021035

BankIt1904654 08PE132 KX021036

BankIt1904654 03PE097 KX021037

BankIt1904654 09PE193 KX021038

BankIt1904654 09PE173 KX021039

BankIt1904654 08PE107 KX021040

BankIt1904654 08PE150 KX021041

BankIt1904654 02PE043 KX021042

BankIt1904654 02PE055 KX021043

BankIt1904654 02PE061 KX021044

BankIt1904654 09PE161 KX021045

BankIt1904654 02PE026 KX021046

BankIt1904654 08PE117 KX021047

BankIt1904654 08PE076 KX021048

BankIt1904654 08PE082 KX021049

BankIt1904654 02PE078 KX021050

BankIt1904654 02PE005 KX021051

BankIt1904654 03PE084 KX021052

BankIt1904654 08PE096 KX021053

BankIt1904654 02PE074 KX021054

BankIt1904654 08PE141 KX021055

BankIt1904654 03PE088 KX021056

BankIt1904654 08PE090 KX021057

BankIt1904654 02PE073 KX021058

BankIt1904654 08PE070 KX021059

BankIt1904654 09PE195 KX021060

BankIt1904654 02PE049 KX021061

BankIt1904654 02PE027 KX021062

BankIt1904654 09PE170 KX021063

BankIt1904654 08PE103 KX021064

BankIt1904654 08PE106 KX021065

BankIt1904654 08PE073 KX021066

BankIt1904654 08PE144 KX021067

BankIt1904654 02PE076 KX021068

BankIt1904654 07PE002 KX021069

BankIt1904654 02PE054 KX021070

BankIt1904654 02PE037 KX021071

BankIt1904654 02PE077 KX021072

BankIt1904654 08PE088 KX021073

BankIt1904654 02PE003 KX021074

BankIt1904654 02PE030 KX021075

BankIt1904654 03PE098 KX021076

BankIt1904654 08PE109 KX021077

BankIt1904654 02PE024 KX021078

BankIt1904654 08PE114 KX021079

BankIt1904654 08PE121 KX021080

BankIt1904654 02PE033 KX021081

BankIt1904654 02PE052 KX021082

BankIt1904654 02PE042 KX021083

BankIt1904654 09PE187 KX021084

BankIt1904654 09PE165 KX021085

BankIt1904654 09PE186 KX021086

BankIt1904654 09PE197 KX021087

BankIt1904654 03PE096 KX021088

BankIt1904654 09PE160 KX021089

BankIt1904654 02PE032 KX021090

BankIt1904654 08PE067 KX021091

BankIt1904654 09PE210 KX021092

BankIt1904654 02PE072 KX021093

BankIt1904654 08PE154 KX021094

BankIt1904654 08PE074 KX021095

BankIt1904654 08PE059 KX021096

BankIt1904654 08PE071 KX021097

BankIt1904654 08PE120 KX021098

BankIt1904654 02PE046 KX021099

BankIt1904654 08PE054 KX021100

BankIt1904654 08PE100 KX021101

BankIt1904654 09PE232 KX021102

BankIt1904654 02PE015 KX021103

BankIt1904654 02PE066 KX021104

BankIt1904654 02PE011 KX021105

BankIt1904654 02PE007 KX021106

BankIt1904654 03PE105 KX021107

BankIt1904654 02PE071 KX021108

BankIt1904654 08PE072 KX021109

BankIt1904654 03PE099 KX021110

BankIt1904654 02PE017 KX021111

BankIt1904654 08PE155 KX021112

BankIt1904654 08PE139 KX021113

BankIt1904654 09PE214 KX021114

BankIt1904654 02PE050 KX021115

BankIt1904654 02PE045 KX021116

BankIt1904654 02PE079 KX021117

BankIt1904654 08PE153 KX021118

BankIt1904654 02PE035 KX021119

BankIt1904654 02PE080 KX021120

BankIt1904654 02PE038 KX021121

BankIt1904654 03PE086 KX021122

BankIt1904654 03PE085 KX021123

BankIt1904654 08PE064 KX021124
